# Branched Poly(Aryl Piperidinium) Membranes for Anion‐Exchange Membrane Fuel Cells

**DOI:** 10.1002/anie.202114892

**Published:** 2021-12-27

**Authors:** Xingyu Wu, Nanjun Chen, Harm‐Anton Klok, Young Moo Lee, Xile Hu

**Affiliations:** ^1^ Laboratory of Inorganic Synthesis and Catalysis (LSCI) Institute of Chemical Sciences and Engineering (ISIC) École Polytechnique Fédérale de Lausanne (EPFL) BCH 3305 Lausanne 1015 Switzerland; ^2^ Department of Energy Engineering College of Engineering Hanyang University Seoul 04763 (Republic of Korea; ^3^ Laboratoire des Polymères Institut des Matériaux and Institut des Sciences et Ingénierie Chimiques École Polytechnique Fédérale de Lausanne (EPFL) Switzerland

**Keywords:** Anion exchange membrane, Branched structure, Durability, Fuel cells, Poly(aryl piperidinium)s

## Abstract

Anion‐exchange membrane fuel cells (AEMFCs) are a promising, next‐generation fuel cell technology. AEMFCs require highly conductive and robust anion‐exchange membranes (AEMs), which are challenging to develop due to the tradeoff between conductivity and water uptake. Here we report a method to prepare high‐molecular‐weight branched poly(aryl piperidinium) AEMs. We show that branching reduces water uptake, leading to improved dimensional stability. The optimized membrane, b‐PTP‐2.5, exhibits simultaneously high OH^−^ conductivity (>145 mS cm^−1^ at 80 °C), high mechanical strength and dimensional stability, good processability, and excellent alkaline stability (>1500 h) in 1 M KOH at 80 °C. AEMFCs based on b‐PTP‐2.5 reached peak power densities of 2.3 W cm^−2^ in H_2_−O_2_ and 1.3 W cm^−2^ in H_2_‐air at 80 °C. The AEMFCs can run stably under a constant current of 0.2 A cm^−2^ over 500 h, during which the b‐PTP‐2.5 membrane remains stable.

## Graphical abstract

High‐molecular‐weight branched poly(aryl piperidinium) membranes with high conductivity (147 mS cm^−1^ at 80 °C) and excellent mechanical (26 % swelling ratio) and chemical stability have been prepared. Anion‐exchange membrane fuel cells (AEMFCs) using these membranes exhibit high peak power density (2.3 W cm^−2^) and durability (500 h).

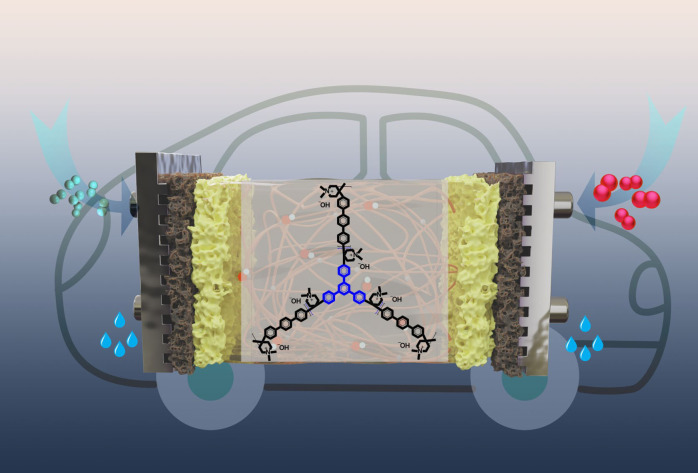

## Introduction

Low‐temperature hydrogen fuel cells are an essential component of hydrogen economy, which has been advocated as a major path towards the decarbonization of the energy sector.[^1^, ^2^, ^3^] Within the two main types of low‐temperature hydrogen fuel cells, anion‐exchange membrane fuel cells (AEMFCs) are potentially more cost‐effective and more scalable than proton‐exchange membrane fuel cells (PEMFCs) because the former may use platinum‐group‐metal (PGM)‐free catalysts, more affordable bipolar plates, and inexpensive, hydrocarbon‐based membranes and ionomers.[^4^, ^5^, ^6^, ^7^] AEMFCs may also offer additional cost saving with simplified cooling system and air loop.^[8]^ However, AEMFCs are still at an early stage of development and many technical targets have not yet been achieved, especially in terms of the power density and durability.[^9^, ^10^, ^11^, ^12^] Anion‐exchange membranes (AEMs) are a key component of AEMFCs that dictate their performance.^[13]^ AEMs are typically produced from polymers containing cationic groups that can transport OH^−^ ions and water molecules. There are several fundamental challenges associated with the development of high‐performance AEMs for AEMFC: i) Most polymers (polyether ether ketone (PEEK), polysulfone (PSF), poly(phenylene oxide) (PPO), polybenzimidazole (PBI)) and cationic groups (ammonium, imidazolium, phosphonium and organometallic cations) tend to degrade under alkaline conditions, especially at 80 °C where AEMFCs operate.[^14^, ^15^, ^16^] ii) The OH^−^ conductivity of AEMs is much lower than the H^+^ conductivity of proton‐exchange membranes (PEMs) at a similar ion exchange capacity (IEC) due to a lower mobility of OH^−^ (about 50 % of H^+^) and incomplete dissociation of quaternary ammonium hydroxide.[^17^, ^18^] As a result, to achieve equivalent ion conductivity to PEMs, AEMs need to possess higher IECs. However, a high IEC leads to high water uptake and swelling, which compromises the mechanical stability of the AEM. The tradeoff of AEMs between ion conductivity and water uptake limit their suitability in AEMFCs.[^19^, ^20^]

**Figure 1:**
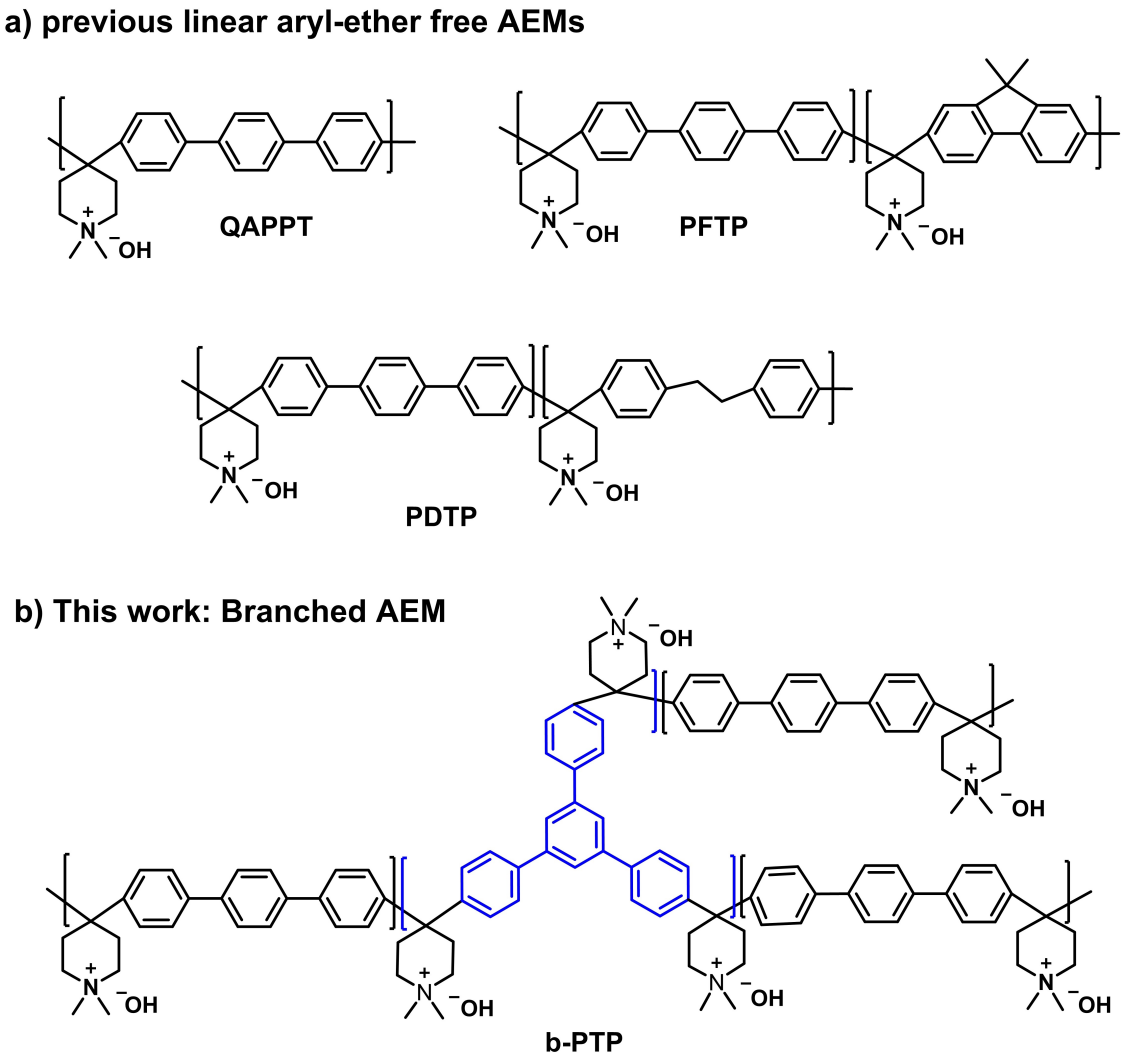
a) Chemical structures of representative examples of previously reported aryl‐ether free AEMs: QAPPT,^[28]^ PDTP^[30]^ and PFTP,^[31]^ b) this work: branched poly(terphenyl piperidinium) AEMs.

In response to the first challenge, aryl‐ether free polymers[^9^, ^21^, ^22^, ^23^, ^24^, ^25^] and piperidinium‐type cationic groups^[26]^ have been developed, exhibiting enhanced alkaline stability. A number of AEMs based on aryl‐ether free polymer backbones and piperidinium groups have been prepared (Figure 1a) and used in AEMFCs, some of which show promising peak power densities (PPD) and durability.[^22^, ^27^, ^28^, ^29^, ^30^, ^31^] The group of Zhuang^[28]^ reported that AEMFCs using quaternary ammonia poly (N‐methyl‐piperidine‐co‐p‐terphenyl) (QAPPT) AEMs had a PPD of 1.45 W cm^−2^ (under O_2_) and 120 h durability under a constant current density of 0.2 A cm^−2^ at 80 °C. The group of Yan^[29]^ synthesized poly(terphenyl piperidinium‐co‐trifluoroacetophenone) AEMs that led to AEMFCs with a PPD of 0.92 W cm^−2^ (under air) and 300 h durability under a constant current density of 0.5 A cm^−2^ at 95 °C. The group of Lee[^30^, ^31^] developed poly(diphenyl‐co‐terphenyl piperidinium) (PDTP) and poly(fluorenyl‐co‐terphenyl piperidinium) (PFAP) AEMs. PDTP‐based AEMFCs exhibited a PPD of 2.58 W cm^−2^ (under O_2_). PFAP‐based AEMFCs exhibited a PPD of 2.34 W cm^−2^ (under O_2_) and 200 h durability under a constant current density of 0.2 A cm^−2^ at 70 °C. Despite this significant progress, durability of AEMs under AEMFC operating conditions need to be further improved for practical applications.[^9^, ^32^]

The poly(aryl piperidinium) (PAP) membranes illustrated in Figure 1a are subject to the same tradeoff between ionic conductivity and water uptake due to the second challenge described above. The group of Jannasch^[27]^ reported that poly(p‐terphenyl N,N‐dimethylpiperidinium) (PTPipQ1) has very high water uptake (145 % at 20 °C) likely due to a low molecular weight. Here, we describe a facile synthetic approach to prepare high‐molecular‐weight branched poly(aryl piperidinium) polymers (b‐PAP) (Figure 1b). The branch structure of the polymers engenders a high rigidity, leading to much reduced water uptake and swelling ratio compared to linear PAPs. The b‐PAP AEMs exhibit all the desired properties for applications in AEMFCs: high conductivity, alkaline stability, and mechanical robustness. Indeed, AEMFCs with the b‐PAP AEM demonstrate state‐of‐the‐art PPD (2.3 W cm^−2^ with O_2_) and exceptionally high operational stability (over 500 h).

## Results and Discussion

### Synthesis and Characterization

**Figure 2:**
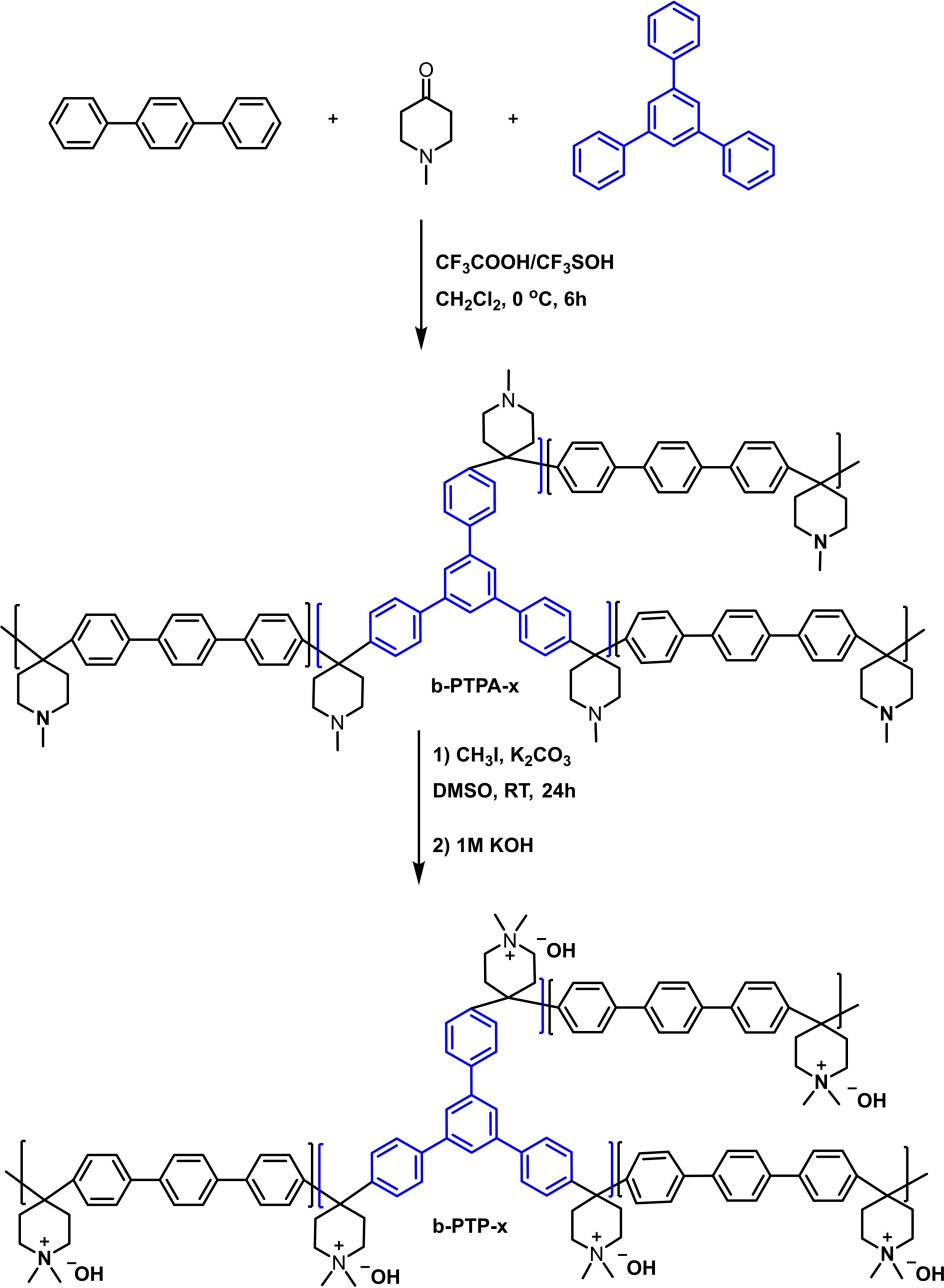
Synthesis of branched poly(terphenyl piperidinium)s.

The polymers were synthesized according to Figure 2. The neutral, branched poly(terphenyl piperidine) (b‐PTPA‐*x* polymers, where *x* is the molar ratio (in percent) of 1,3,5‐triphenylbenzene (TPB) to the aryl monomers), were prepared by a trifluoromethanesulfonic acid (TFSA) catalyzed polycondensation reaction of p‐terphenyl (TP), TPB, and N‐methyl‐4‐piperidone.^[33]^ To obtain a high molecular weight, the polymerization conditions including the ratio of TFSA to piperidone, monomer concentration, and reaction times were systematically varied and optimized (Table S1). The cationic, branched poly(terphenyl piperidinium) (b‐PTP‐*x*) polymers were prepared from b‐PTPA‐*x* and iodomethane via the Menshutkin reaction. The lack of a suitable calibration standard, a strong adsorption of the cationic poly(aryl piperidinium) polymers on chromatography columns, and the poor solubility of the neutral poly(aryl piperidine) polymers (generally insoluble in water, THF, DCM and DMF) make it difficult to measure their molecular weights by gel permeation chromatography/size‐exclusion chromatography (GPC/SEC). Thus, we used intrinsic viscosity [η] in DMSO as an indicator of their molecular weights, which was also used in previous studies.[^27^, ^29^, ^30^, ^31^] The optimized conditions were: a TFSA/piperidone ratio of 10, a monomer (piperidone) concentration of 0.45 mol L^−1^ and a reaction time of 6 h (Table S1, entry 5, for *x*=2.5). All b‐PTPA‐*x* (*x*=1, 2.5 and 5) polymers were synthesized under these conditions, with yields higher than 85 %.

The linear PTP reference polymer that was obtained by polymerization of TP and N‐methyl‐4‐piperidone had a viscosity of 2.84 dL g^−1^ at 22 °C. Introducing a small amount of TPB comonomer (1 mol%, for b‐PTP‐1) increased largely the intrinsic viscosity to 5.52 dL g^−1^. The b‐PTP‐2.5 polymer had an even higher viscosity of over 6 dL g^−1^. Further increasing the percentage of TPB to 5 %, however, led to a polymer (b‐PTP‐5) with reduced solubility. The viscosity of b‐PTP‐2.5 is higher than all previously reported PAPs (Table S2).[^27^, ^29^, ^30^, ^31^, ^34^] For comparison, the PTPipQ1 polymer developed by Jannasch and co‐workers^[27]^ had a viscosity of only 0.39 dL g^−1^ at 30 °C due to its low molecular weight. The dynamic light scattering (DLS) spectra of b‐PTP‐*x* (Figure S1) are consistent with a higher molecular weight upon branching. These data confirm our method as a highly efficient strategy to prepare high‐molecular‐weight PAPs.

**Figure 3:**
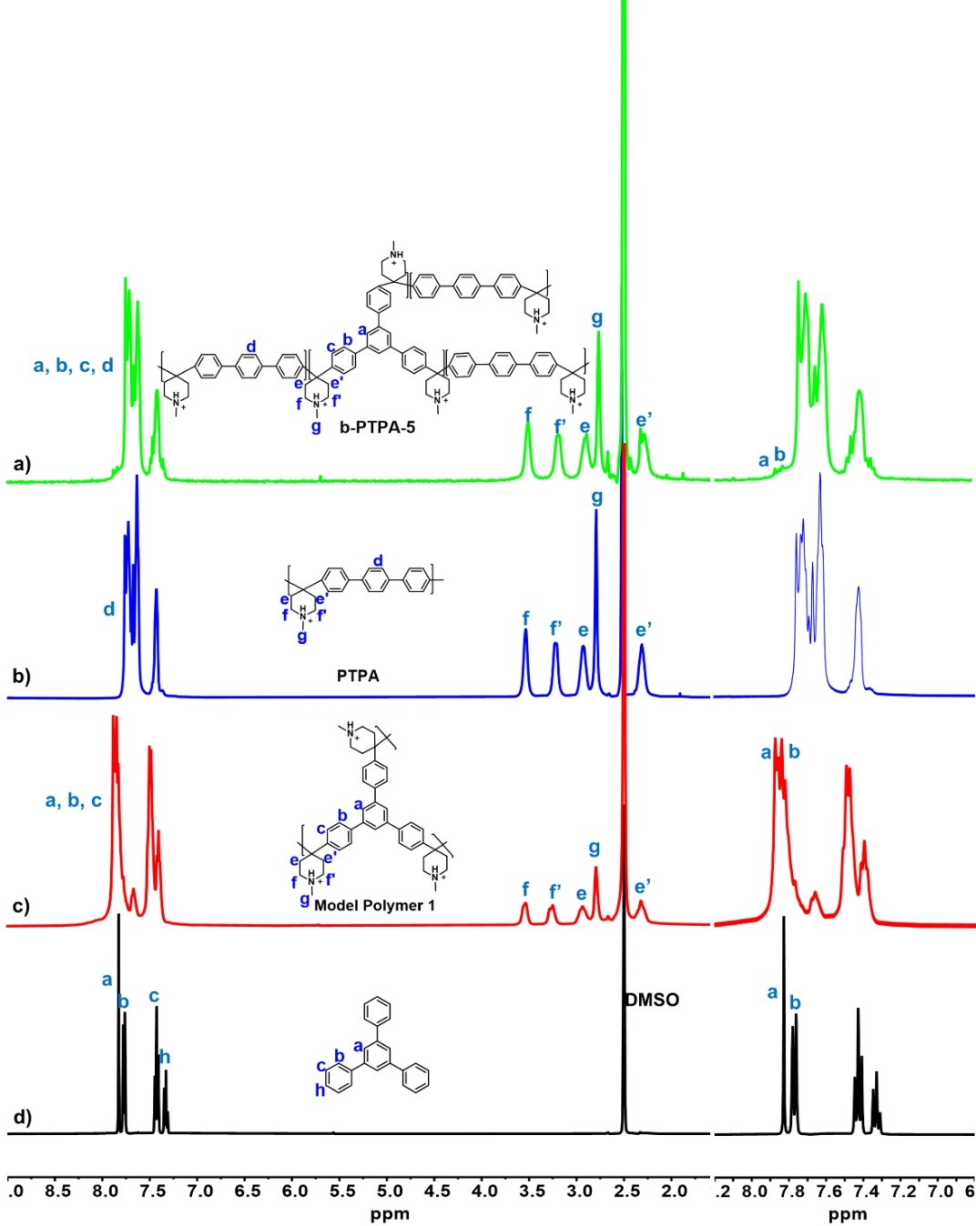
^1^H NMR spectra of a) b‐PTPA‐5, b) PTPA, c) model polymer **1** and d) 1, 3, 5‐triphenyl benzene using with 5 % trifluoroacetic acid (TFA) in DMSO‐*d*_6_ as solvents.

The molecular structures of b‐PTPA‐*x* were confirmed by ^1^H NMR spectroscopy. In the ^1^H NMR spectrum of b‐PTPA‐5, three small peaks between 7.8 and 8 ppm were assigned to a and b hydrogens of TPB (Figure 3a). To support this assignment, three model polymers were synthesized. The peaks from a and b hydrogens of model polymer 1, hyperbranched poly(triphenyl benzene piperidine), were shifted to higher position compared to 1,3,5‐triphenyl benzene (Figure 3a and b). This shift is similar to that of b‐PTPA‐5. Similarly, in the spectra of model polymer 2, branched poly(biphenyl piperidine, and model polymer 3, branched poly(benzene piperidine, the peaks from a and b hydrogens were observed in the range of 7.8–8 ppm (Figure S2 and S3). These NMR spectra confirmed the branching structure of the polymers. In addition, when the percentage of TPB in the polymer was increased from 2.5 to 5, the signals of the two peaks in the range of 7.8–8 ppm increased (Figure S4), consistent with their assignments to a and b hydrogens. Quaternization did not change the polymer structure, as indicated by the ^1^H NMR spectrum of b‐PTP‐*x* (Figure S5). Mohr titration showed that the experimental IECs of b‐PTP‐*x* were in agreement with the theoretical values, indicating a full conversion of the quaternization reactions (Table 1).

**Table 1:** IEC, water uptake (WU), swelling ratio (SR), conductivity and hydration number of b‐PTP‐*x* AEMs.

AEMs	IEC [mmol g^−1^]		WU [%]		SR [%]		*σ* [mS cm^−1^]		*λ*	
	Theoretical	Titration	40 °C	80 °C	40 °C	80 °C	40 °C	80 °C	40 °C	80 °C
PTP	2.80	2.84	94.3	109.8	30.7	33.2	81.4	137.7	18.4	21.5
b‐PTP‐1	2.80	2.80	91.2	107.3	25.0	30.4	84.9	138.6	18.1	21.3
b‐PTP‐2.5	2.81	2.84	69.3	79.5	22.7	25.9	87.1	146.7	13.6	15.6
b‐PTP‐5	2.81	2.87	85.2	98.4	28.5	32.5	82.2	136.2	16.5	19.0

### Mechanical Property, Conductivity and Dimensional Stability

**Figure 4:**
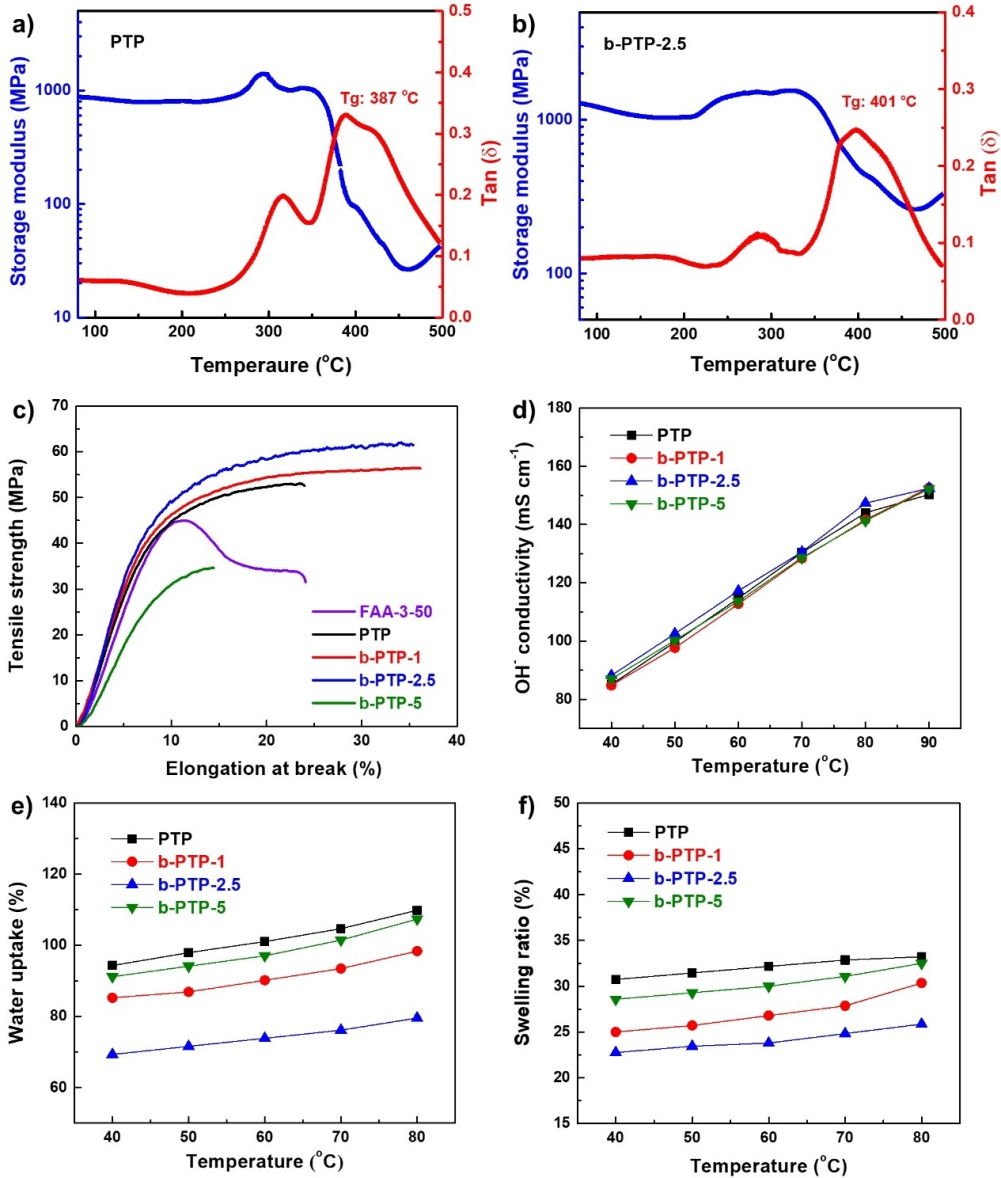
a) Storage modulus and tan *δ* of a) PTP and b) b‐PTP‐2.5 membranes, c) mechanical properties of b‐PTP‐*x* (I^−^ form) and commercial FAA‐3‐50 (Cl^−^ form) in the dry state, d) OH^−^ conductivity of b‐PTP‐*x* AEMs at different temperatures and 100 % RH, e) water uptake and f) swelling ratio of b‐PTP‐*x* AEMs.

Thermogravimetric analysis (TGA) indicates that the b‐PTP‐*x* membranes are thermally stable until 200 °C (Figure S6). Dynamic mechanical analysis (DMA) was applied to measure the glass transition temperature (*T*_g_) of b‐PTP‐2.5 and PTP (Figure 4a and b). For the b‐PTP‐2.5 polymer a *T*_g_ of 401 °C was measured, which was slightly higher than the *T*_g_ of the linear PTP sample (387 °C). Moreover, the b‐PTP‐2.5 membrane exhibits a higher storage modulus (over 1300 MPa vs. 860 MPa at 80 °C) than the PTP reference membrane, indicating the former's excellent dynamic mechanical properties. The b‐PTP‐*x* (*x*=1, 2.5) membranes have higher tensile strength and elongation at break than the linear PTP reference membrane (Figure 4c). Increasing *x* to 5 led to a polymer with reduced solubility so that the resulting membrane is rough and fragile. The b‐PTP‐2.5 membrane has the highest tensile strength (62 MPa) and elongation at break (36 %) among all samples (b‐PTP‐*x*, PTP, and a commercial FAA‐3‐50 membrane).

The b‐PTP‐*x* membranes exhibit high OH^−^ conductivity of more than 80 mS cm^−1^ at 40 °C and more than 135 mS cm^−1^ at 80 °C (Figure 4d and Table 1). Branching and its degree have a small effect in the conductivity, and the b‐PTP‐2.5 membrane is most conductive (87 mS cm^−1^ at 40 °C and 147 mS cm^−1^ at 80 °C). This level of conductivity is close to that of Nafion, the benchmark PEM (187 mS cm^−1^),^[35]^ and it is considered sufficient for practical applications.^[36]^

The b‐PTP‐*x* AEMs have high IEC values (≈2.8 mmol g^−1^), which are the same as the linear PTP reference membrane (Table 1). Generally, AEMs with IEC values over 2.0 mmol g^−1^ have high water uptake, leading to poor dimensional stability and mechanical properties.[^13^, ^21^] For example, the linear PTP reference membrane had water uptake of 94 % and 110 %, swelling ratio of 31 % and 33 % and hydration number of 18.4 and 21.5 at 40 °C and 80 °C, respectively. Branching significantly reduced the water uptake, swelling ratio and hydration number. The best sample is b‐PTP‐2.5, whose water uptake and swelling ration are 28 % and 22 % lower than PTP at 80 °C (Figure 4e, f, and Table 1). The water uptake and swelling ratio of b‐PTP‐2.5 only increased 15 % and 14 %, respectively, as the temperature increased from 40 °C to 80 °C. These increases are much smaller than those of conventional AEMs such as PPO and PBI. The limited increases observed here might be due to the hydrophobicity of poly(aryl piperidinium)s and the high molecular weight of the present polymers.

The above data indicated the b‐PTP‐2.5 membrane as the best sample among the b‐PTP‐*x* series, so this membrane was chosen for further characterization and application. The b‐PTP‐2.5 membrane is highly processable, as thin membranes with a thickness down to 20 μm and a size over 200 cm^2^ can be easily casted. This membrane has excellent flexibility and remains intact after 5 cycles of kneading and recovering. (Figure S7) In fuel cells, undesirable H_2_ diffusion from anode to cathode can reduce fuel efficiency, decrease cathode potential, and form aggressive peroxide radicals.^[37]^ Gratifyingly, the b‐PTP‐2.5 membrane exhibits a lower H_2_ permeability (18 Barrer, 1 Barrer=10^−10^ cm^3^ (STP) cm cm^−2^ s^−1^ cm Hg^−1^) compared to Nafion 212 (85 Barrer) and FAA‐3‐50 (35 Barrer) (Figure S8).

### Alkaline Sability

**Figure 5:**
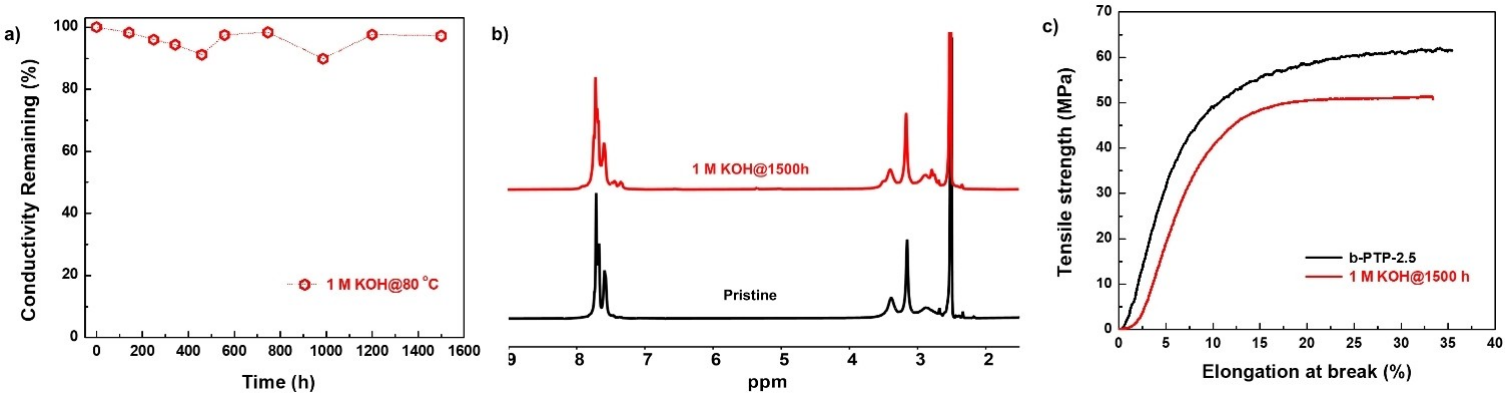
Alkaline stability of the b‐PTP‐2.5 membrane in 1 M KOH at 80 °C. a) Remaining hydroxide conductivity at different interval time, b) ^1^H NMR spectra and c) mechanical properties before and after 1500 h alkaline treatment.

The ex situ alkaline stability of the b‐PTP‐2.5 membrane was investigated by monitoring the variations of surface morphology, mechanical property, chemical structure and conductivity after alkaline treatment. After 1500 h in 1 M KOH at 80 °C, no cracks or holes could be observed from the digital photo and microscopy (Figure S9 and S10). The membrane maintained its high hydroxide conductivity after being soaked 1500 h in 1 M KOH at 80 °C (Figure 5a). No chemical degradation of the b‐PTP‐2.5 membrane was detected via ^1^H NMR spectroscopy (Figure 5b and Figure S10). The treated b‐PTP‐2.5 membrane possessed similar mechanical property as the pristine sample (tensile strength of 52 MPa and elongation at break of 33 %) (Figure 5c). These data indicate excellent alkaline stability of b‐PTP‐2.5. An accelerated degradation test was also conducted on b‐PTP‐2.5 by soaking it in a concentrated KOH solution (3 M) at 80 °C. The b‐PTP‐2.5 membrane lost 20 % of its initial conductivity after 1500 h (Figure S11), indicating partial degradation under these harsh conditions No cracks or holes could be observed in the film after the test, but its tensile strength and elongation at break decreased by 21 % and 58 %, respectively (Figure S9 and Figure S11). ^1^H NMR spectra indicate that the dimethyl piperidinium groups started to decompose after 986 h due to Hofmann β‐elimination^[27]^ (Figure S12). After 1500 h, about 17 % dimethyl piperidinium groups were degraded. Although an accelerated stability test in highly concentrated KOH at an elevated temperature could reveal the stability limit of AEMs, the chemical and mechanical environments of AEMs in AEMFCs under operating conditions are different from those of the test. As we shown below, stability test in operating AEMFCs is more relevant.

### Fuel Cell Performance and Durability

**Figure 6:**
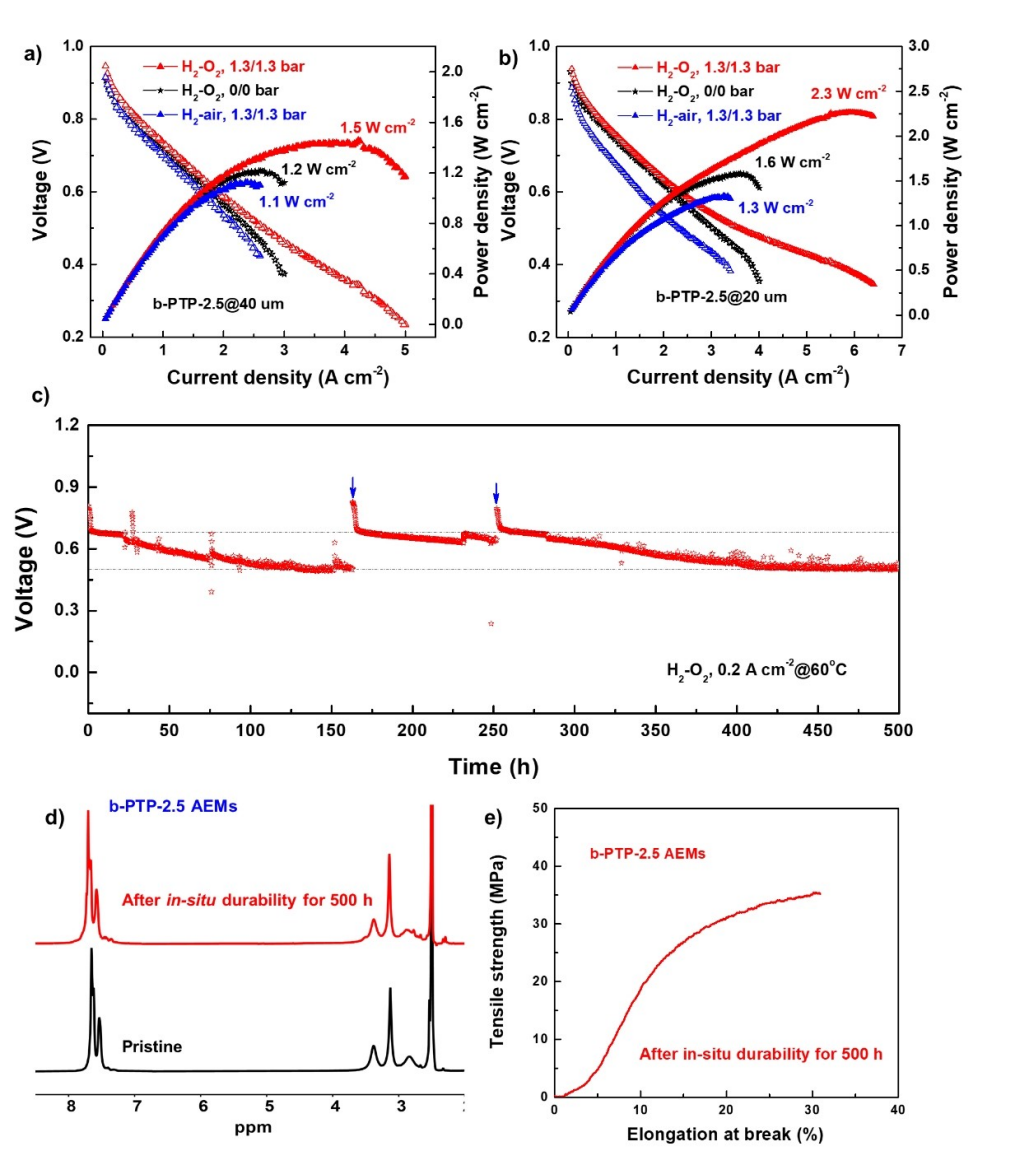
AEMFC performance based on a b‐PTP‐2.5 AEM with a thickness of a) 40 μm and b) 20 μm. Testing conditions: 0.6 mg cm^−2^ Pt−Ru/C anode with additional carbon, 0.4 mg cm^−2^ Pt/C cathode, 1000/1000 mL min^−1^ A/C H_2_−O_2_ (CO_2_‐free air) flow rate, 75 %/100 % A/C RH, 80 °C; c) AEMFC durability test under a constant current density of 0.2 A cm^−2^ at 60 °C; d) ^1^H NMR spectra and e) mechanical properties of b‐PTP‐2.5 membrane (OH^−^ form) after the durability test for 500 h.

The b‐PTP‐2.5 membranes with two thicknesses (40 μm and 20 μm) were used to make MEAs via a catalyst‐coated membrane method, using benchmark Pt‐based catalysts (See methods). Using H_2_‐O_2_ and under 75 %/100 % A/C RH and 0/0 A/C back pressure (A=anode; C=cathode; RH=relative humidity), the PPD of AEMFC increased from 1.2 W cm^−2^ to 1.6 W cm^−2^ at 80 °C when the membrane thickness was reduced from 40 μm to 20 μm (Figure 6a and b). The thinner membrane led to a better performance, which is attributed to a lower cell resistance and improved water diffusion between anode and cathode.[^23^, ^29^] A significant improvement of PPDs was obtained when applying 1.3/1.3 bar back pressure to accelerating electrode reactions and optimizing water management. The AEMFC with the 20 μm b‐PTP‐2.5 membrane achieved an impressive PPD of 2.3 W cm^−2^. Using H_2_‐air (CO_2_ free), the PPDs of AEMFCs reached 1.1 and 1.3 W cm^−2^ for 40 μm and 20 μm b‐PTP‐2.5 membrane, respectively. The PPDs of AEMFCs based on commercial AEMs and polyaromatics membranes are typically less than 1 W cm^−2^.[^20^, ^31^, ^38^, ^39^, ^40^] Although comparison of PPDs of AEMFCs in the literature is not straightforward due to the use of different catalysts, cell configuration, and operating conditions, the fact that the present AEMFCs have one of the topmost PPDs. Table S3[^23^, ^28^, ^29^, ^30^, ^31^, ^41^, ^42^, ^43^] is a testimony of the superior properties of the b‐PTP‐2.5 membranes under fuel cell operating conditions.

Figure 6c shows the in situ durability test of AEMFCs under a constant current density of 0.2 A cm^−2^ at 60 °C in H_2_−O_2_ with A/C dew points of 53 °C/60 °C. Instant jumps of discharging voltage were observed (indicated by the black arrows), which implied the water droplets formed at the anode (flooding process) were swiped out by O_2_ gas. Within the initial 160 h durability test, the voltage decreased from 0.68 V to 0.51 V at 110 h and stabilized at 0.51 V for 50 h. To probe the origin of the voltage loss, the catalyst layers of the MEA were removed and fresh catalysts were sprayed onto the same membrane for further durability test (refreshment process, indicated by blue arrows). The voltage of refreshed cell jumped back to 0.68 V, indicating the intactness of the b‐PTP‐2.5 membrane after durability test. To further prove this, a second refreshment process was applied at 250 h. The voltage jumped back to 0.68 V again. Finally, the present AEMFCs based on b‐PTP‐2.5 membrane can be operated stably at 0.51 V under a 0.2 A cm^−2^ current density over 500 h. Note that the present AEMFC was fullly operational and the b‐PTP‐2.5 membrane was not damaged after 500 h. In fact, the cell reached a steady state at this point. To the best of our knowledge, this is the first time an AEMFC based on a PAP‐type AEM exhibits in situ durability longer than 500 h. AEMFCs based on commercial FAA membranes showed significant voltage loss within 48 h.^[31]^ The group of Zhuang^[28]^ presented QAPTP‐based AEMFCs with 120 h durability at 0.2 A cm^−2^. The group of Yan^[29]^ reported PAP‐TP‐*x*‐based AEMFCs with 300 h durability at 0.5 A cm^−2^. The group of Lee[^30^, ^31^] reported PDTP‐based AEMFCs with 100 h durability at 0.4 A cm^−2^ and PFAP‐based AEMFCs with 200 h durability at 0.2 A cm^−2^. Similar to our b‐PTP membranes, PDTP and PFAP membranes are based on poly(aryl piperidinium)s, but with a linear instead of branched structure. These three membranes are expected to have a similar chemical stability, and hence ex situ alkaline stability. However, the branched structure in b‐PTP leads to a higher molecular weight (viscosity of 6.18 dL g^−1^ for b‐PTP‐2.5 vs. 4.5 dL g^−1^ for PDTP‐25 and 4.08 dL g^−1^ for PFTP‐13), which results in higher dimensional and mechanical stability. The latter should be the main origin of the higher durability of b‐PTP‐based AEMFCs. If high‐molecular‐weight linear poly(aryl piperidinium)s can be prepared, we expect them to have similar in situ stability profiles to the present b‐PTP membranes under fuel cell operating conditions.

The possible changes of surface morphology, chemical structure, and mechanical properties of the b‐PTP‐2.5 membrane after 500 h in situ durability test were probed. After the test, the b‐PTP‐2.5 membrane remained robust and flexible. No cracks or hole could be observed from microscopy (Figure S9). ^1^H NMR spectrum indicated no degradation of the membrane (Figure 6d). Importantly, the b‐PTP‐2.5 membrane (in OH^−^ form) maintained high tensile strength (35 MPa) and elongation at break (30 %) after the 500 h cell test (Figure 6e).

## Conclusion

In summary, we have developed a facile synthetic approach to branched poly(aryl piperidinium) AEMs. The optimized membrane, b‐PTP‐2.5, exhibits simultaneously high OH^−^ conductivity (>145 mS cm^−1^ at 80 °C), reduced water uptake and swelling ratio, and desirable mechanical properties (tensile strength >60 MPa and elongation at break >35 %). The membrane is flexible and easy to process. This membrane is intact after being immersed in 1 M KOH for 1500 h at 80 °C. AEMFCs based on this membrane achieve PPDs of up to 2.3 W cm^−2^ in H_2_−O_2_ and up to 1.3 W cm^−2^ in H_2_–air, which are among the highest values known for AEMFCs. These AEMFCs can run stably over 500 h, and the b‐PTP‐2.5 membrane remains stable during this process. Our work demonstrates branched poly(terphenyl piperidinium) AEMs as promising membranes for applications in AEMFCs. The strategy of branching might be applicable to the development of other types of AEMs.

## Conflict of interest

X. Wu and X. Hu are inventors of a European Priority Patent Application on branched anion exchange membranes.

1

## Supporting information

As a service to our authors and readers, this journal provides supporting information supplied by the authors. Such materials are peer reviewed and may be re‐organized for online delivery, but are not copy‐edited or typeset. Technical support issues arising from supporting information (other than missing files) should be addressed to the authors.

Supporting Information

## Data Availability

The data that support the findings of this study are available in the Supporting Information of this article. Datasets are also available on Zenodo: https://doi.org/10.5281/zenodo.5779052).
