# Supplementary material for: Branched Poly(Aryl Piperidinium) Membranes for Anion‐Exchange Membrane Fuel Cells
Source: Angew Chem Int Ed Engl. 2021 Dec 27;61(7):e202114892. doi: 10.1002/anie.202114892 (PMC9304273; doi:10.1002/anie.202114892)
Supplement: Supplementary file 1 — Supporting Information [file ANIE-61-0-s001.pdf]

Angewandte Chemie
Eine Zeitschrift der Gesellschaft Deutscher Chemiker

Supporting Information

Branched Poly(Aryl Piperidinium) Membranes for Anion-Exchange Membrane Fuel Cells

X. Wu, N. Chen, H.-A. Klok, Y. M. Lee, X. Hu**

Table of Contents

Experimental Procedures 3

Materials 3

Synthesis of branched poly(terphenyl piperidine) (b-PTPA-x) 3

Synthesis of model polymer 1: hyperbranched poly(triphenylbenzene piperidine) 3

Synthesis of model polymer 2: branched poly(benzene piperidine) 3

Synthesis of model polymer 3: branched poly(biphenyl piperidine) 3

Synthesis of branched poly(terphenyl piperidinium) (b-PTP-x) 3

Membrane preparation 3

Intrinsic viscosity 4

1H nuclear magnetic resonance spectroscopy 4

Scanning electron microscopy 4

Thermal and mechanical properties 4

Dynamic mechanical analysis 4

IEC measurement 5

Water uptake and swelling ratio 5

Conductivity measurement 5

Gas permeability 5

Alkaline stability 6

Fuel cell performance 6

Results and Discussion 7

References 22

Author Contributions 23

Experimental Procedures

Materials

Para-Terphenyl (TP), N-methyl-4-piperidone, iodomethane, benzene, potassium carbonate (K_2CO_3) and Dimethyl sulfoxide (DMSO) were purchased from Sigma-Aldrich. 1,3,5-Triphenylbenzene (TPB) was purchased from abcr GmbH (Germany). Biphenyl (BP) was purchased from Acros Organics. Trifluoromethanesulfonic acid (TFSA) was purchased from Fluorochem. Trifluoroacetic acid (TFA) was purchased from TCI. Dichloromethane (DCM) and ethyl acetate were purchased from Thermo Fisher Scientific Inc. (Suisse). All chemicals were used directly without further purification.

Synthesis of branched poly(terphenyl piperidine) (b-PTPA-x)

The typical synthetic procedure of b-PTPA-x is as follows (using b-PTP-1 as an example). In a flask, TP (3.408g, 0.99 equiv.), TPB (0.046 g, 0.01 equiv.), and N-methyl-4-piperidone (1.70 g, 1.005 equiv.) were added into DCM (17 mL). The solution was stirred with an overhead agitator under 0 ^oC for 30 min. Then TFA (1.22 mL, 1.1 equiv.) and TFSA (13.3 mL, 10.05 equiv.) were added dropwise to the solution. During the reaction, the color of the solution changed from red to dark green. After 6 h, the resulting viscous solution was poured into excess amount of methanol and the precipitated white polymer fibers were cut into small pieces. These pieces were collected by filtration and washed with 1M K_2CO_3 at 50 ^oC overnight, followed by washing three time with deionized (DI) water and dried in oven at 80 ^oC under vacuum for 24 h.

Synthesis of model polymer 1: hyperbranched poly(triphenylbenzene piperidine)

In a flask, TPB (4.696 g, 1 equiv.) and N-methyl-4-piperidone (1.734 g, 1 equiv.) were added into DCM (17.45 mL). The solution was stirred with overhead agitator under 0 ^oC for 30 min. Then TFA (1.22 mL, 1.1 equiv.) and TFSA (13.61 mL, 10 equiv.) were added dropwise to the solution. During reaction, the color of the solution changed from light yellow to brick red. The resulted viscous solution was poured into excess amount of methanol. The white product was filtered and washed with 1M K_2CO_3 at 50 ^oC overnight, followed by washing three time with water and dried in oven at 80 ^oC under vacuum for 24 h. The obtained Model Polymer 1 is soluble in DMF, NMP, THF and $CHCl_3$.

Synthesis of model polymer 2: branched poly(benzene piperidine)

In a flask, benzene (1.109 g, 0.95 equiv.), TPB (0.229 g, 0.05 equiv.) and 1-methyl-4-piperidone (1.734 g, 1.025 equiv.) were added into DCM (17.45 mL). The solution was stirred with overhead agitator under 0 ^oC for 30 min. Then TFA (1.22 mL, 1.1 equiv.) and TFSA (13.61 mL, 10.25 equiv.) were added dropwise to the solution. The resulted viscous solution was poured into excess amount of methanol. The white product was filtered and washed with 1M K_2CO_3 at 50 ^oC overnight, followed by washing three time with water and dried in oven at 80 ^oC under vacuum for 24 h. The obtained model polymer 2 is soluble in NMP, THF and $CHCl_3$.

Synthesis of model polymer 3: branched poly(biphenyl piperidine)

In a flask, BP (2.190 g, 0.95 equiv.), TPB (0.229 g, 0.05 equiv.) and N-methyl-4-piperidone (1.734 g, 1.025 equiv.) were added into DCM (17.45 mL). The solution was stirred with overhead agitator under 0 ^oC for 30 min. Then TFA (1.22 mL, 1.1 equiv.) and TFSA (13.61 mL, 10.05 equiv.) were added dropwise to the solution. During reaction, the color of the solution changed from light yellow to brick red. The resulted viscous solution was poured into excess amount of methanol. The white product was filtered and washed with 1M K_2CO_3 at 50 ^oC overnight, followed by washing three time with water and dried in oven at 80 ^oC under vacuum for 24 h.

Synthesis of branched poly(terphenyl piperidinium) (b-PTP-x)

In a flask, b-PTPA-x (1 g) was suspended in DMSO (30 mL).and the solution was stirred at room temperature for 30 min. Subsequently, K_2CO_3 (0.39 g) and iodomethane (1 mL) were added and the reaction was kept stirred at room temperature for 24 h in the dark. Ethyl acetate was added to the resulting viscous solution. The light-yellow precipitate was filtered, washed three times with water, and dried in oven at 80 ^oC under vacuum for 24 h.

Membrane preparation

b-PTP-x (1 g) was dissolved in 30 mL DMSO and the polymer solution was filtered through a 0.45 µm polytetrafluoroethylene (PTFE) filter and casted onto a clean glass plate. Subsequently, the solution was evaporated at 80 °C for 12 h, 120 °C for 12 h, and dried at 120 °C under vacuum for 24 h to completely remove the residual solvent. The membrane in I^- form was peeled off from glass plate. Membrane in Br^- form was obtained by ion exchanging in 1 M KBr solution at 80 °C for 12 h, followed by washing with deionized water for 3 times to remove residual salts. Membrane in OH^- form was obtained by ion exchanging in 1 M KOH solution at 80 °C for 12 h, followed by washing with degassed deionized water for 3 times under N_2 atmosphere. Membrane in OH^- form was stored under N_2 to avoid contamination by CO_2 and the formation of carbonate.

Intrinsic viscosity

The intrinsic viscosity ([η]) was determined by using an Ubbelohde viscometer. b-PTP-x was dissolved in DMSO into four different concentrations. The efflux time of each concentration was recorded four times at 22 °C. The reduced viscosity (η_{red}) and inherent viscosity (η_{inh}) were calculated by:

$$\eta_{red} = \frac{\frac{t_s}{t_b} - 1}{c} \quad (1)$$

$$\eta_{inh} = \frac{\ln(\frac{t_s}{t_b})}{c} \quad (2)$$

where t_b (s) is the efflux time of DMSO, t_s (s) is the efflux time of the polymer solution at concentration c (g dL^{-1}). The intrinsic viscosity ([η]) was obtained by extrapolating η_{red} and η_{inh} to c=0 and the average value was adopted.

^{1}H nuclear magnetic resonance spectroscopy

The ^{1}H spectra of protonated b-PTPA-x and model polymers, as well as the b-PTP-x, were measured by a Bruker Avance 400 spectrometer using DMSO-d_6 with 5% vol% TFA. By adding TFA, the peak of water was shifted so that the peaks of piperidine/piperidinium ring could be observed.

Scanning electron microscopy

Surface morphology of dry b-PTP-x membranes in I^- form was observed with a Zeiss Merlin microscope at 1 kV and equipped with an Inlens secondary electron detector. Membrane samples were coated with gold before analysis.

Thermal and mechanical properties

The thermal properties of b-PTPA-1 powder, protonated b-PTPA-1 powder, b-PTP-1 (I^- form) powder, as well as b-PTP-x membranes (I^- form) were measured by a Perkin Elmer TGA 4000 under N_2 atmosphere. Samples were hold at 120 °C for 10 min to remove adsorbed water, and then heated from 50 °C to 800 °C with a heating rate of 10 °C min^{-1}.

The mechanical properties of b-PTP-x membranes were measured by Universal Testing Machine (UTM; AGS-500NJ, Shimadzu, Tokyo, Japan) at room temperature. Membrane samples were cut into a dumbbell-like shape with effective area of 2 mm × 10 mm and stretched at 1 mm min^{-1}.

Dynamic mechanical analysis

The storage modulus and tan δ of PTP and b-PTP-2.5 membranes in I^- form were measured by a dynamic thermomechanical analysis (DMA, Q800. TA instrument, DE, USA) system. Membrane samples were cut into 9 mm × 20 mm rectangle shape and then measured with a preload force of 0.01 N and a force track of 125% under N_2 atmosphere. The sample was ramped at 4 °C min^{-1} until 450 °C. The peak of tan δ stands for the glass transition temperature (T_g) of membrane sample.

IEC measurement

The ion exchange capacity (IEC) of b-PTP-x membranes was measured by Mohr titration. Weights (W_{dry}) of membrane samples in Br^- form were recorded after drying in an oven under vacuum at 80 °C overnight. Then, the samples were ion exchanged with 0.1 M

$NaNO_3$ at 50 °C for 12 h and repeated three times. The solutions were combined and titrated by 0.01 M $AgNO_3$ using K_2CrO_4 as indicator. The IEC (mmol g^{-1}) of b-PTP-x membranes in Br^- and OH^- form can be calculated by:

$$\mathrm{IEC}(Br^-) = \frac{0.01 * V_{AgNO_3}}{W_{dry}} \quad (3)$$

$$\mathrm{IEC}(OH^-) = \frac{0.01 * V_{AgNO_3}}{W_{dry} - 0.629 * V_{AgNO_3}} \quad (4)$$

where V_{AgNO3} is the volume (mL) of consumed $AgNO_3$ solution.

Water uptake and swelling ratio

Dry weight (W_{dry}) and length (L_{dry}) of b-PTP-x membranes samples were recorded after drying in an oven at 80 °C under vacuum. Then the samples were immersed in degassed deionized water for 12 h at different temperatures under N_2. The wet weight (W_{wet}) and length (L_{wet}) of the samples were recorded after wiping excessive water from the surface. The water uptake (*WU*) and swelling ratio (*SR*) can be calculated by:

$$WU(\%) = \frac{W_{wet} - W_{dry}}{W_{dry}} \times 100\% \quad (5)$$

$$SR(\%) = \frac{L_{wet} - L_{dry}}{L_{dry}} \times 100\% \quad (6)$$

The hydration number (λ) is the number of adsorbed water molecules per piperidinium group. It can be calculated by:

$$\lambda = \frac{1000 * WU}{IEC(OH^-) * M(H_2O)} \quad (7)$$

where $M(H_2O)$ is the relative molecular weight of water (18 g mol^{-1}).

Conductivity measurement

The in-plane ion conductivity (mS cm^{-1}) of b-PTP-x membranes was measured by an Autolab PGSTAT302N equipped with a Scribner 740 MTS. Membrane samples in 10 mm × 30 mm were assembled into a four-electrode cell and tested using the alternative current (AC) impedance in the frequency range of 1 Hz to 0.1 MHz. The assembly and measurement were operated under N_2 to avoid CO_2 contamination. The measurements were carried out at different temperature at 100% RH. The ion conductivity σ (mS cm^{-1}) of membrane samples can be calculated by:

$$\sigma = \frac{L}{AR} \quad (8)$$

where *L* (cm) is the distance between working and counter electrode, *A* (cm^2) is the cross-sectional area calculated by the thickness (cm) and width (cm) of the membrane samples, *R* (kΩ) is the Ohm impedance obtained from electrochemical impedance spectra.

Gas permeability

The H_2 permeabilities of PTP, b-PTP-2.5, commercial FAA-3-50 and Nafion 212 membranes were measured by a gas permeability testing system equipped with gas chromatography (GC, 490 Micro GC, Agilent Technologies, USA) and two mass flowmeters (MFC, M3030V, Line Tech, Korea).[1, 2] The measurements were conducted under different RHs with 2.2 bar unilateral backpressure at 80 °C. Gas permeability *P* (Barrer, 1 Barrer = 10^{-10} cm^3 (STP) cm cm^{-2} s^{-1} $cmHg^{-1}$) can be calculated by:

$$P = \frac{V M_{gas} d}{P_{feed} R T A \rho} \frac{d_p}{d_t} \quad (9)$$

where *V* (cm^3), M_{gas} (g mol^{-1}), *d* (um) and P_{feed} (760 mmHg) are the volume of measurement cell, molecular weight of permeating gas, membrane thickness and the pressure of feed gas, and *R* (L mmHg K^{-1} mol^{-1}), *T* (K), *A* (cm^2) and *ρ* (g cm^{-3}) are the gas constant, testing temperature, effective area of samples and the density of permeating gas, respectively. $\frac{d_p}{d_t}$ denotes the change rate of permeated gas pressure over time.

Alkaline stability

The alkaline stability of b-PTP-2.5 membrane was measured by immersing into 1 M and 3 M KOH at 80 °C over 1500 h. The KOH solution was refreshed weekly. Before testing the conductivity, the sample was washed three times with degassed deionized water under N_2. To measure ^1H NMR spectra, surface morphology and mechanical property, the sample was ion exchanged with 1M KI at 80 °C for 12 h, followed by washing three times with deionized water and drying.

Fuel cell performance

The single fuel cell performance was measured by a fuel cell station (CNL, Seoul, Republic of Korea). The anode slurry was prepared by adding 12.5 mg Pt-Ru/C (40 wt% Pt, 20 wt% Ru, Hispec, Alfa Aesar, USA) and 5 mg poly(fluorenyl-co-biphenyl piperidinium) (PFBP, 5 wt% in DMSO) ionomers to isopropanol/water (1.2 mL/0.12 mL) solution. The composition of ionomer: carbon: Pt-Ru is 1: 75: 1.5. The cathode slurry was prepared by adding 12.5 mg Pt/C (40 wt% Pt, Hispec, Alfa Aesar, USA) and 3.75 mg PFBP ionomer to isopropanol/water (1 mL/0.1 mL) solution. The composition of ionomer: carbon: Pt is 1: 2: 1.33. The slurries were sonicated for 45 min at 0 °C before spraying. Then, the anode and cathode slurries were sprayed onto both sides of b-PTP-2.5 membranes (I^- form) along with metal catalyst loadings of 0.6 and 0.4 mg cm^{-2}, respectively. The prepared catalyst-coated membrane (CCM) was immersed in 1 M NaOH at room temperature for 12 h, followed by washing three times in deionized water. After that, the CCM was assembled with gas diffusion layers (GDLs), PTFE gaskets and graphite bipolar plates into single cell at a torque of 60 in-lb. The effective area of the cell is 5 cm^2.

The assembled cell was connected with gas lines and heating system. After the temperature of humidifier and gas lines reached the set values, cell temperature was increased to 70 °C. The cell was activated at a constant voltage of 0.5 V at flowrate of H_2 and O_2 at 1000 mL min^{-1} until no fluctuation of current density. After that, the temperature of cell was increased to 80 °C and the polarization curve was recorded.

The *in-situ* durability of fuel cell was measured under a constant current density of 0.2 A cm^{-2} at 60 °C with 400/400 mL min^{-1} H_2/O_2 flowrate with Anode (A)/Cathode (C) dew points of 53 °C and 60 °C. The cell was refreshed two times during durability test. After durability test, the membrane was disassembled from the MEA and used for ^1H NMR, surface morphology and mechanical property measurements.

Results and Discussion

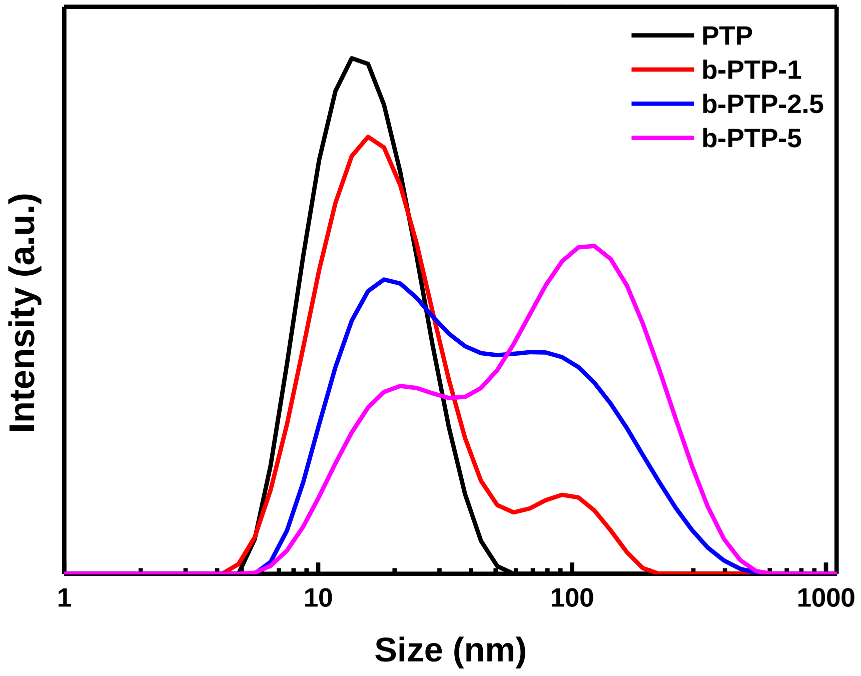

“

Figure S1. DLS intensity-based size distribution for PTP and b-PTP-x.

The linear PTP reference exhibited one DLS peak at around 14 nm. This peak was shifted to higher size and its intensity was decreased with increasing branching degree. Meanwhile, another peak at around 100 nm appeared for the branched polymers. The size and intensity of the latter peak increased with increasing branching degree. Although the interpretation of the DLS spectra of b-PTP-x is difficult, the data are consistent with a higher molecular weight upon branching.

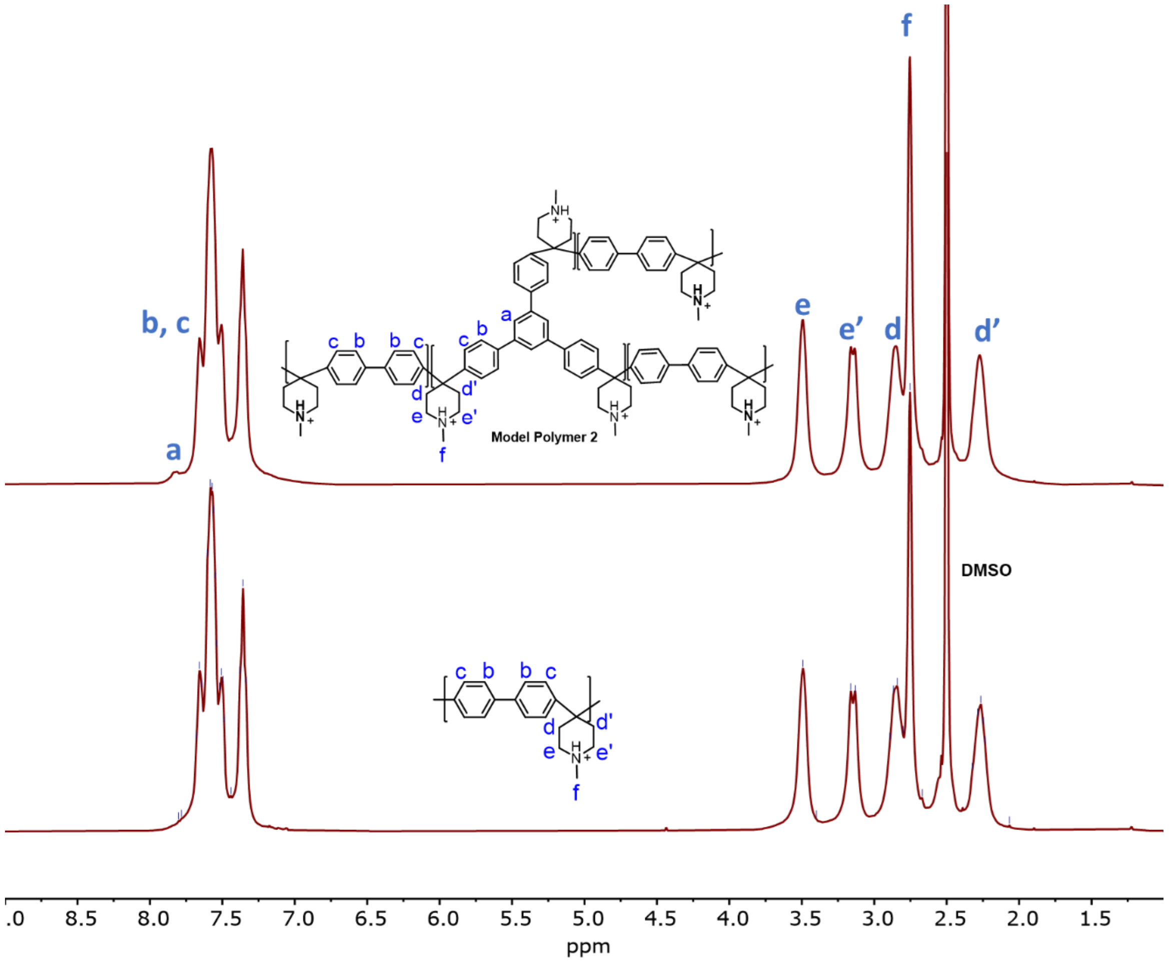

Figure S2 ^{1}H NMR spectra of model polymer 2 and its linear reference in DMSO-d_6 with 5% TFA. TFA can improve the solubility by protonating the amine groups. Meanwhile, TFA shifts the water peak from 3.3 ppm to 12-14 ppm.

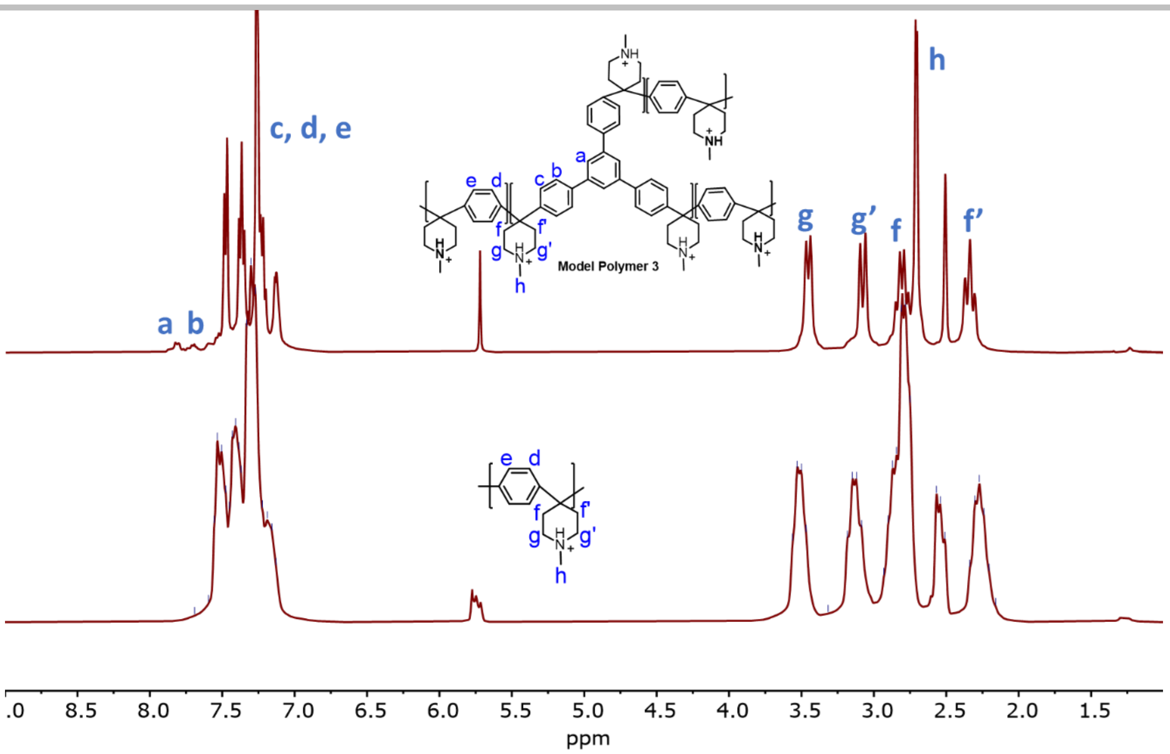

Figure S3 ^{1}H NMR spectra of model polymer 3 and its linear reference in DMSO-d_6 with 5% TFA. TFA can improve the solubility by protonating the amine groups. Meanwhile, TFA shifts the water peak from 3.3 ppm to 12-14 ppm.

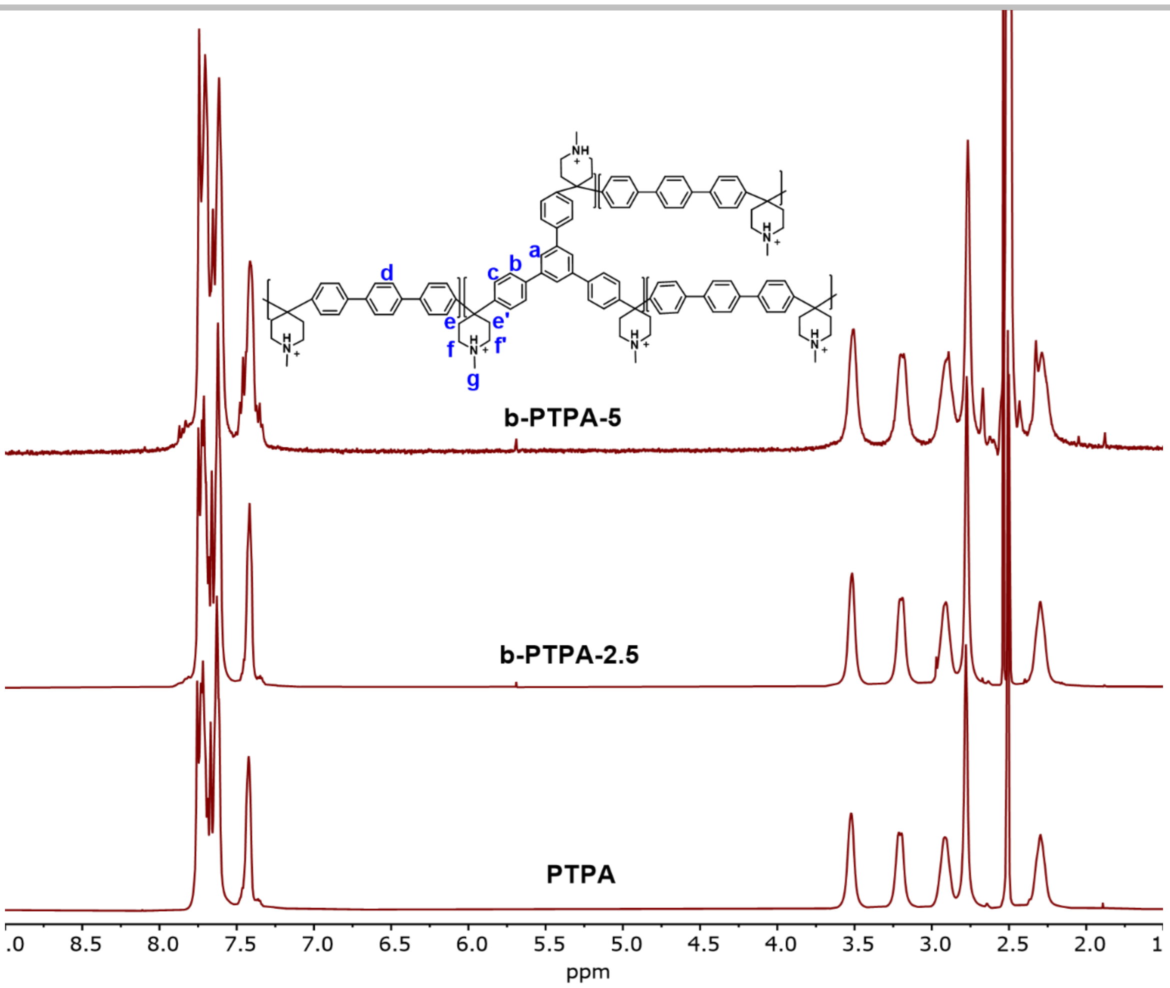

Figure S4 ^{1}H NMR spectra of PTPA, b-PTPA-2.5 and b-PTPA-5 in DMSO-d_6 with 5% TFA. TFA can improve the solubility by protonating the amine groups. Meanwhile, TFA shifts the water peak from 3.3 ppm to 12-14 ppm.

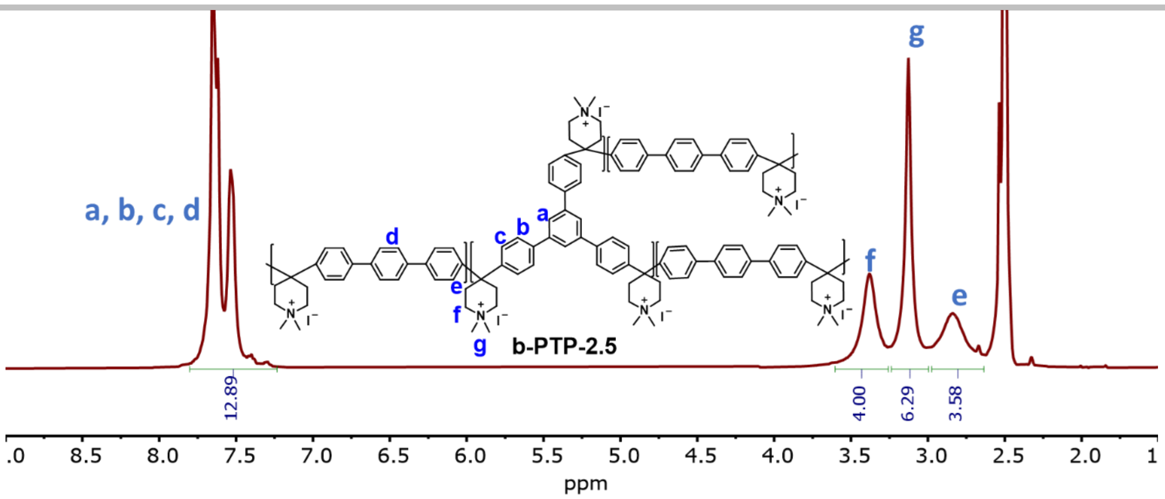

Figure S5 ^{1}H NMR spectrum of b-PTP-2.5 in DMSO-d_6 with 5% TFA. TFA can improve the solubility by protonating the amine groups. Meanwhile, TFA shifts the water peak from 3.3 ppm to 12-14 ppm.

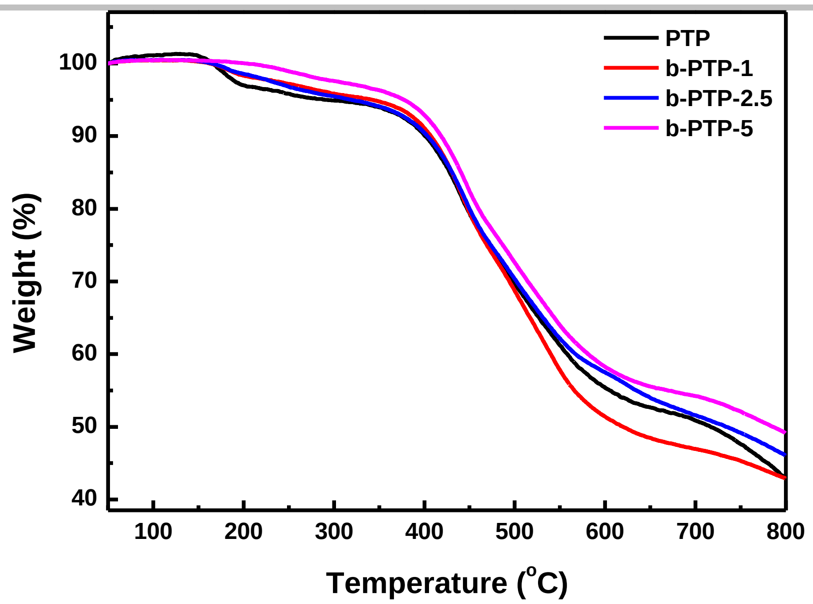

Figure S6 TGA curves of b-PTP-x AEMs in OH^- form under N_2 atmosphere.

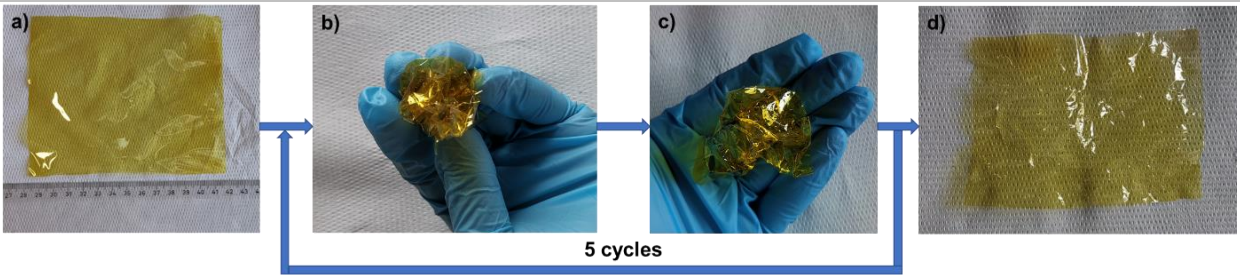

Figure S7 Mechanical toughness test of b-PTP-2.5 in I^- form (20 μm). a) fresh membrane, b) and c) kneaded membrane, d) recovered membrane.

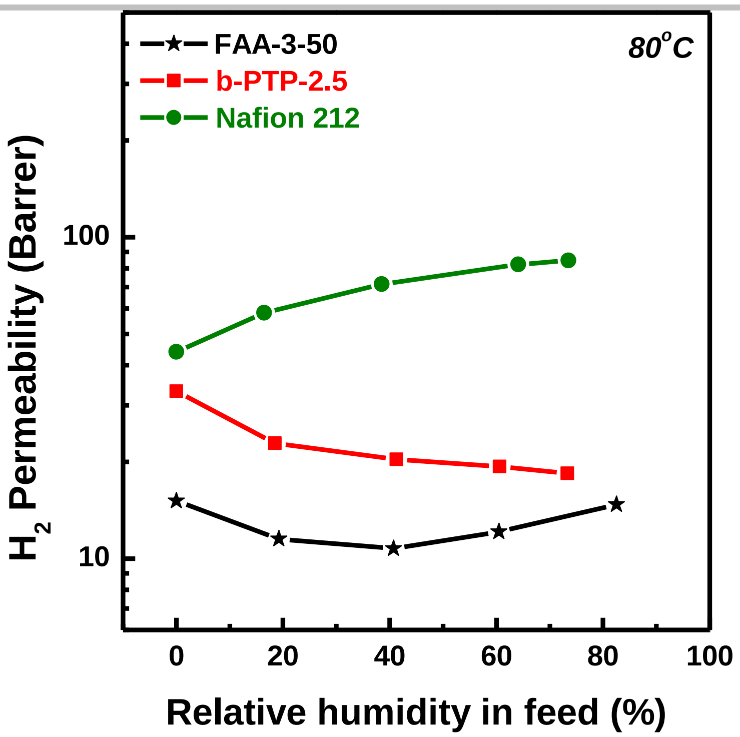

Figure S8 H_2 permeability of b-PTP-2.5 membrane (I^- form) at different RH and 80 ºC. The values of commercial FAA-3-50 and Nafion 212 membranes are presented for comparison.

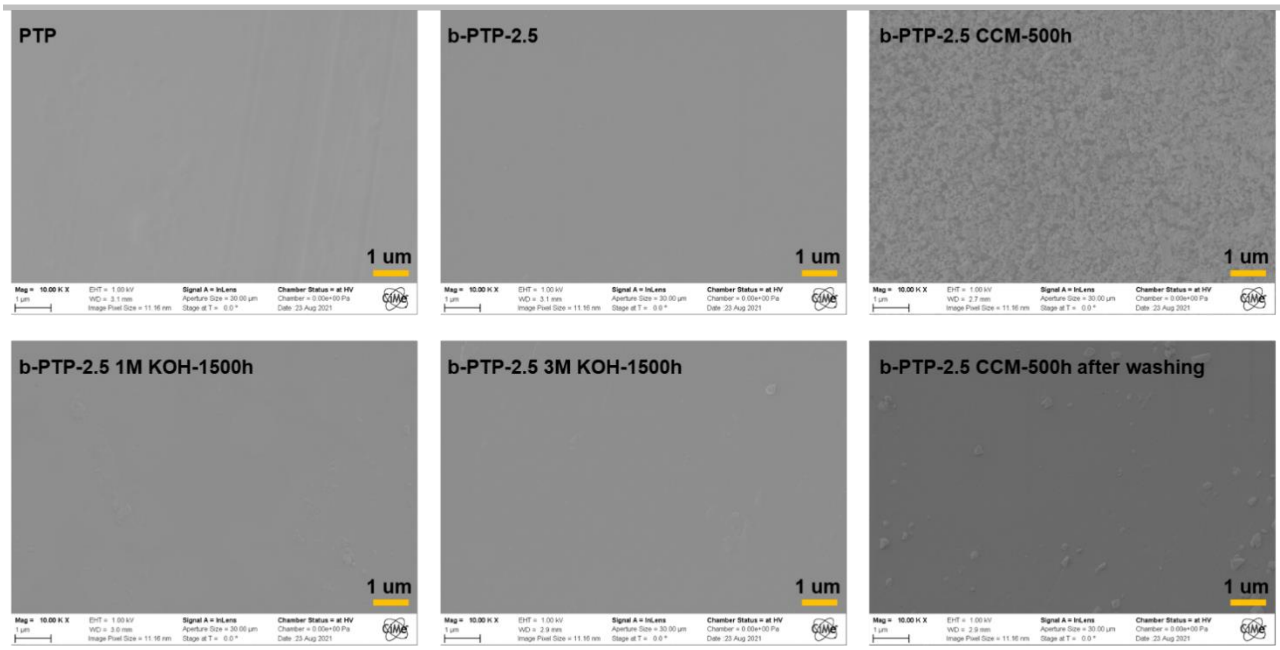

Figure S9 Surface morphology of PTP reference membrane, b-PTP-2.5 membrane, b-PTP-2.5 membrane after a 500 h in-situ durability test, b-PTP-2.5 membrane after a 1500 h treatment in 1 M and 3 M KOH at 80 ^{o}C.

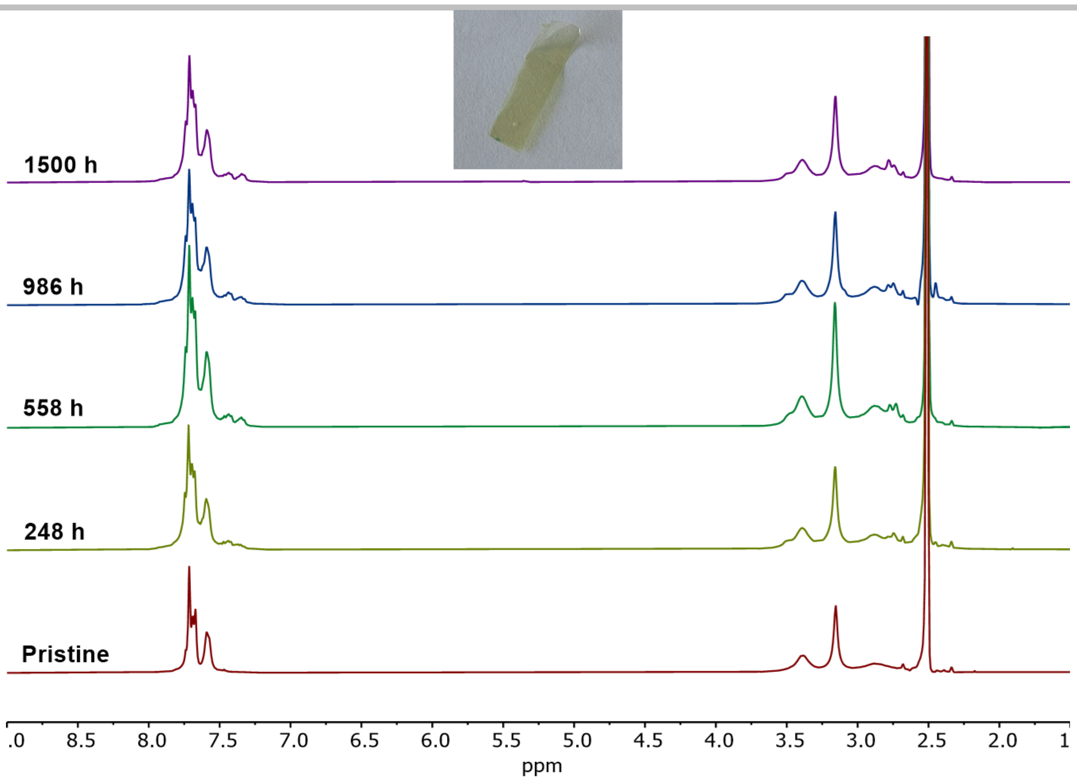

Figure S10 ^{1}H NMR spectra of b-PTP-2.5 membrane after immersing in 1 M KOH at 80 ^{o}C for different durations.

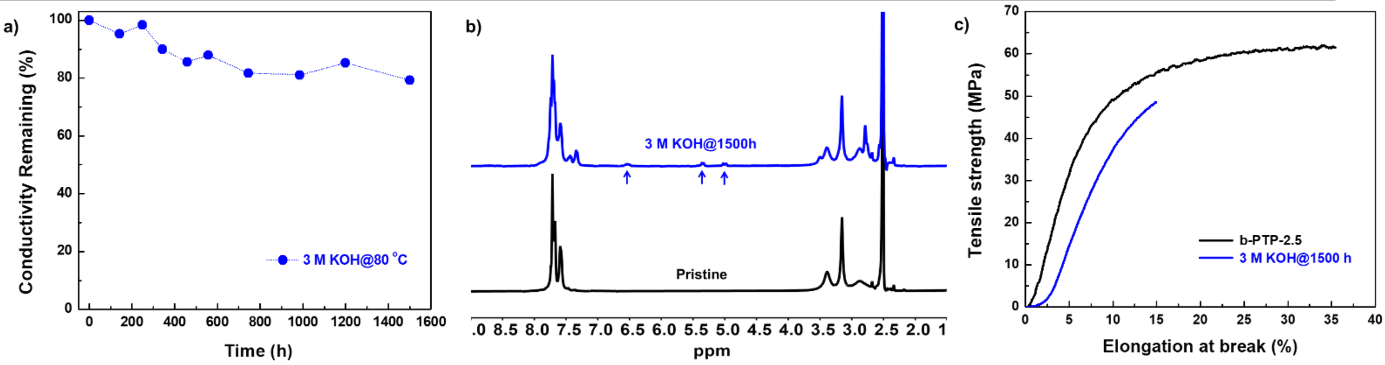

Figure S11 Alkaline stability of the b-PTP-2.5 membrane in 3M KOH at 80 ºC. a) remaining hydroxide conductivity at different immersing time, b) ^{1}H NMR spectra and c) mechanical properties before and after 1500 h alkaline treatment.

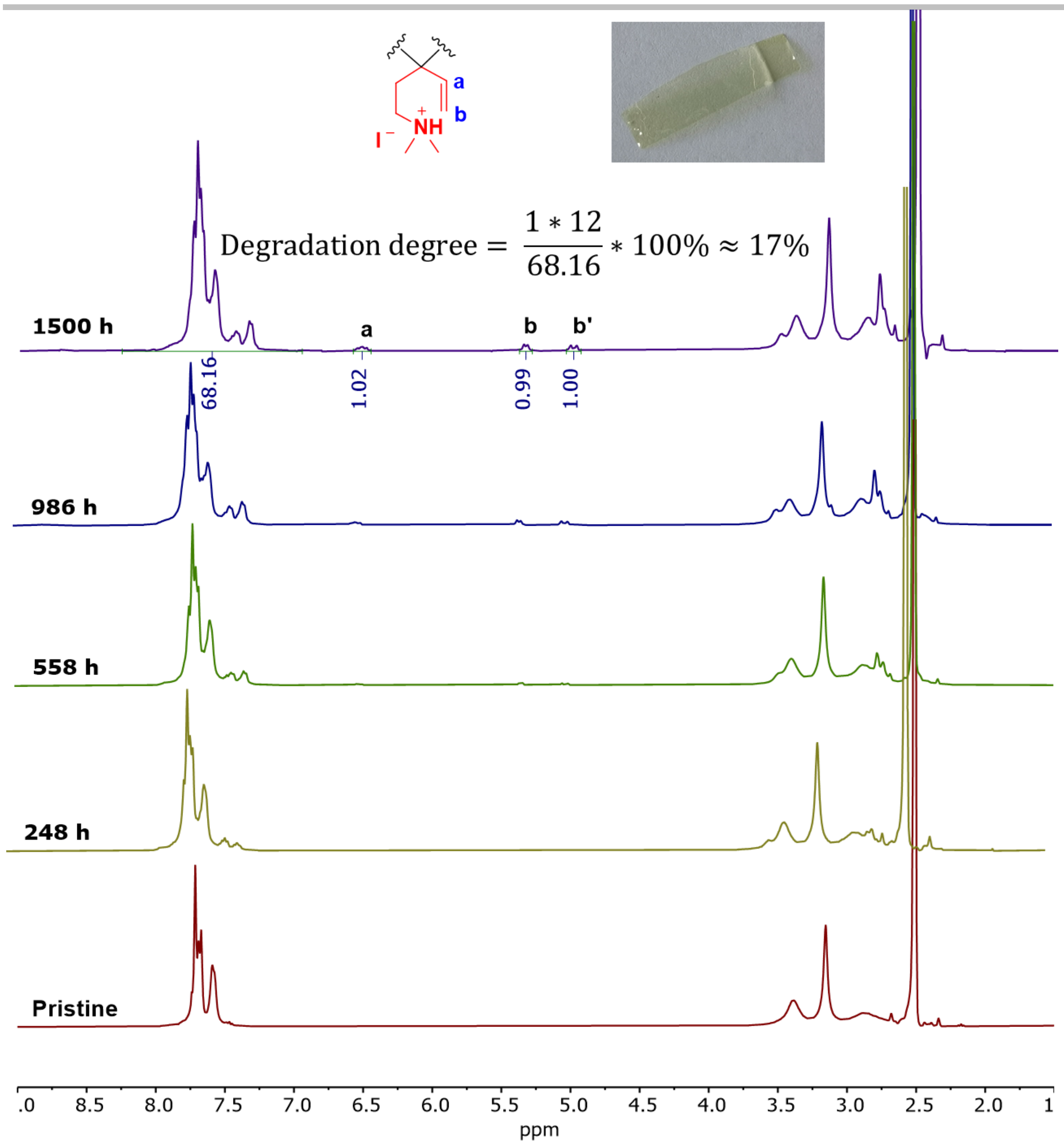

Figure S12 ^{1}H NMR spectra of b-PTP-2.5 membrane after being immersed in 3 M KOH at 80 ^oC for different durations.

$$\text{Degradation degree} = \frac{[Area\ of\ Proton\ b]*12}{[Area\ of\ Aromatic\ protons]} * 100\%$$

Table S1 Polymerization conditions of b-PTPA-x[a].

Entry	TFSA: Piperidone mol/mol	Monomer Conc. mol L^{-1}	TP: TPB: Piperidone	Reaction Time, h	Yield	Viscosity dL g^{-1}
1	6.1	0.45	100:0:100	6	-	-
2	10	0.45	100:0:100	6	91.4%	2.84
3	10	0.1	97.5:2.5:101.25	6	48.5%	1.73
4	10	0.3	97.5:2.5:101.25	6	92.8%	2.67
5	10	0.45	97.5:2.5:101.25	6	89.2%	6.18
6	10	0.6	97.5:2.5:101.25	6	92.5%	4.46
7	10	0.45	97.5:2.5:101.25	24	90.8%	Not Measurable
8	10	0.45	99:1:100.5	6	91.5%	5.52
9	10	0.45	95:5:102.5	6	86.2%	Not measurable

[a]X is the molar ratio (in percent) of 1, 3, 5-triphenyl benzene to the aryl monomers

Table S2 Comparison of viscosity among reported poly(aryl piperidinium)s.

Polymers	Viscosity (dL g^{-1})	Ref.
b-PAP-2.5	6.18	This work
PFTP-13	4.08	[1]
PDTP-75	5	[2]
PAP-TP-x	4.71	[3]
PTPipQ1	0.39 (30 ^oC)	[4]
PBPA	2.18 (30 ^oC)	[5]

Table S3 Summary of AEMs, ionomers, anode and cathode catalysts, PPDs, and durability of AEMFCs

AEMs	Ionomers	Anode catalyst	Cathode catalyst	PPD ($W\ cm^{-2}$)	durability	Ref.
b-PTP-2.5	PFBP-14	Pt-Ru/C (0.60 mg cm^{-2})	Pt/C (0.40 mg cm^{-2})	2.3	>500 h	this work
FAA-3-50[a]	Fumion	Pt/C (0.33 mg cm^{-2})	Pt/C (0.33 mg cm^{-2})	0.3-0.4	<48 h	[1]
PFTP-13[b]	PFBP-14	Pt-Ru/C (0.42 mg cm^{-2})	Pt/C (0.33 mg cm^{-2})	2.34	200 h	[1]
PDTP-25[b]	PFBP-14	Pt-Ru/C (0.39 mg cm^{-2})	Pt/C (0.26 mg cm^{-2})	2.58	100 h	[2]
QAPPT[b]	QAPPT	Pt/C (0.4 mg cm^{-2})	Pt/C (0.4 mg cm^{-2})	1.45	120 h	[6]
PAP-TP-85[b]	PAP-BP-100	Pt/C (< 0.15 mg cm^{-2})	Ag/C (1 mg cm^{-2})	0.92	300 h	[3]
TPN[b]	FLN-55	Pt-Ru/C (0.50 mg cm^{-2})	Pt/C (0.60 mg cm^{-2})	1.46	350 h	[7]
BTMA-HDPE[c]	BTMA-ETFE	Pt-Ru/C (0.70 mg cm^{-2})	Pt/C (0.60 mg cm^{-2})	2.35	1000 h	[8, 9]
HTMA-DAPP[c]	FLNs	Pt-Ru/C (0.75 mg cm^{-2})	Pt/C (0.60 mg cm^{-2})	0.54	933 h	[10]
GT64-15[c]	GTXX	Pt-Ru/C (0.70 mg cm^{-2})	Pt/C (0.60 mg cm^{-2})	3.2	2000 h	[11]
PX75-T50[d]	-	Pt-Ru/C (0.50 mg cm^{-2})	Pt/C (0.50 mg cm^{-2})	0.73	400 h	[12]
TMImPPO[d]	CBQPPO	Pt-Ru/C (0.50 mg cm^{-2})	Pt/C (0.50 mg cm^{-2})	1.37	<48 h	[13]
Other PPO series[d]	Other PPO series	Pt/C (0.4-0.5 mg cm^{-2})	Pt/C (0.4-05 mg cm^{-2})	<0.4	-	[14, 15]
HMT-PMBI[d]	HMT-PMBI	Pt/C (0.4 mg cm^{-2})	Pt/C (0.4 mg cm^{-2})	0.37	110 h	[16]

[a]commercial AEMFCs membranes and ionomers;
[b]PAP-family AEMs and ionomers;
[c]state-of-the-art AEMFCs with durability > 1000 h;
[d]poly(phenylene oxide) (PPO) and polybenzimidazole (PBI) membranes and ionomers

References

[1] J. Choi, M.-H. Kim, J. Y. Han, J. E. Chae, W. H. Lee, Y. M. Lee, S. Y. Lee, J. H. Jang, J. Y. Kim, D. Henkensmeier, *J. Membr. Sci.* **2018**, *568*, 67-75.
[2] C. H. Park, S. Y. Lee, D. S. Hwang, D. W. Shin, D. H. Cho, K. H. Lee, T.-W. Kim, T.-W. Kim, M. Lee, D.-S. Kim, *Nature* **2016**, *532*, 480-483.
[3] N. Chen, H. H. Wang, S. P. Kim, H. M. Kim, W. H. Lee, C. Hu, J. Y. Bae, E. S. Sim, Y.-C. Chung, J.-H. Jang, *Nat. Commun* **2021**, *12*, 1-12.
[4] N. Chen, C. Hu, H. H. Wang, S. P. Kim, H. M. Kim, W. H. Lee, J. Y. Bae, J. H. Park, Y. M. Lee, *Angew. Chem. Int. Ed.*. **2021**, *133*, 7789-7797.
[5] J. Wang, Y. Zhao, B. P. Setzler, S. Rojas-Carbonell, C. B. Yehuda, A. Amel, M. Page, L. Wang, K. Hu, L. Shi, *Nat. Energy* **2019**, *4*, 392-398.
[6] J. S. Olsson, T. H. Pham, P. Jannasch, *Adv. Funct. Mater.* **2018**, *28*, 1702758.
[7] W.-H. Lee, A. D. Mohanty, C. Bae, *ACS Macro Lett.* **2015**, *4*, 453-457.
[8] H. Peng, Q. Li, M. Hu, L. Xiao, J. Lu, L. Zhuang, *J. Power Sources* **2018**, *390*, 165-167.
[9] S. Maurya, S. Noh, I. Matanovic, E. J. Park, C. N. Villarrubia, U. Martinez, J. Han, C. Bae, Y. S. Kim, *Energy Environ. Sci* **2018**, *11*, 3283-3291.
[10] X. Peng, D. Kulkarni, Y. Huang, T. J. Omasta, B. Ng, Y. Zheng, L. Wang, J. M. LaManna, D. S. Hussey, J. R. Varcoe, *Nat. Commun* **2020**, *11*, 1-10.
[11] L. Wang, X. Peng, W. E. Mustain, J. R. Varcoe, *Energy Environ. Sci* **2019**, *12*, 1575-1579.
[12] D. P. Leonard, S. Maurya, E. J. Park, L. D. Manriquez, S. Noh, X. Wang, C. Bae, E. D. Baca, C. Fujimoto, Y. S. Kim, *J. Mater. Chem. A.* **2020**, *8*, 14135-14144.
[13] N. Ul Hassan, M. Mandal, G. Huang, H. A. Firouzjaie, P. A. Kohl, W. E. Mustain, *Adv. Energy Mater.* **2020**, *10*, 2001986.
[14] Y. Kim, Y. Wang, A. France-Lanord, Y. Wang, Y.-C. M. Wu, S. Lin, Y. Li, J. C. Grossman, T. M. Swager, *J. Am. Chem. Soc.* **2019**, *141*, 18152-18159.
[15] X. Liang, M. A. Shehzad, Y. Zhu, L. Wang, X. Ge, J. Zhang, Z. Yang, L. Wu, J. R. Varcoe, T. Xu, *Chem. Mater.* **2019**, *31*, 7812-7820.
[16] L. Zhu, J. Pan, Y. Wang, J. Han, L. Zhuang, M. A. Hickner, *Macromolecules* **2016**, *49*, 815-824.
[17] N. Chen, C. Long, Y. Li, C. Lu, H. Zhu, *ACS Appl. Mater. Interfaces* **2018**, *10*, 15720-15732.
[18] A. G. Wright, J. Fan, B. Britton, T. Weissbach, H.-F. Lee, E. A. Kitching, T. J. Peckham, S. Holdcroft, *Energy Environ. Sci* **2016**, *9*, 2130-2142.

Author Contributions

X. Wu and X. Hu conceived the idea. X. Wu synthesized and characterized the polymer and membrane. N. Chen tested the dynamic mechanical analysis, Gas permeability, mechanical properties, fuel cell performance and in-situ durability. X. Wu and X. Hu wrote the paper, with the support from other co-authors. All authors analyzed and discussed the data. X. Hu and Y.M. Lee supervised the work.
